# Trend of caesarean deliveries in Egypt and its associated factors: evidence from national surveys, 2005–2014

**DOI:** 10.1186/s12884-017-1591-2

**Published:** 2017-12-13

**Authors:** Rami H. Al Rifai

**Affiliations:** Institute of Public Health, College of Medicine and Health Sciences, United Arab Emirates University, Al-Ain, United Arab Emirates

**Keywords:** Egypt, Caesarean section delivery, Maternal health, Maternal delivery

## Abstract

**Background:**

The continued rise in caesarean section (c-section) deliveries raises a major public health concern worldwide. This study assessed the trend of c-section deliveries and examined factors associated with a rise in c-section deliveries among the Egyptian mothers, from 2005 to 2014, by place of delivery.

**Methods:**

This study utilized the 2005, 2008, and 2014 Egypt Demographic and Health Surveys (EDHS). The EDHS reported on the mode of delivery for the last birth occurred within five years preceding each survey including place of delivery and sociodemographic information for a total sample of over 29,000 mothers in the three surveys. To document trend of c-section, the EDHS-2005 was set as a reference in two binary logistic regression models; among all mothers together and for mothers stratified by place of delivery (public or private). *P*-value for the trend was assessed by entering the year of the survey as a continuous variable. The study followed STROBE statement in reporting observational studies.

**Results:**

Institutional-based c-sections increased by 40.7 points from EDHS-2005 to EDHS-2014 (aOR, 3.46, 95%CI: 3.15–3.80, *P*_*trend*_ < 0.001). Compared to mothers with low socioeconomic status (SES), mothers with high SES had higher odds (aOR, 1.78, 95%CI: 1.25–2.54, *P* = 0.001) for c-section, but only in EDHS-2005. The adjusted trend of c-sections was found to be 4.19-time (95%CI: 3.73–4.70, *P <* 0.001) higher in private sector while that in public sector it was 2.67-time (95%CI: 2.27–3.13, *P =* 0.001) higher, in EDHS-2014 relative to EDHS-2005. This increase in the private sector is explained by significant increases among mothers who are potentially at low risk for c-sections; mothers aged 19-24 years vs. ≥35 years (aOR: 0.31, 95%CI: 0.21–0.45, in EDHS-2005 vs. 0.43, 95%CI: 0.33–0.56, in EDHS-2014, *P* < 0.001); primigravida mothers vs. mothers with ≥4 children (aOR: 1.62, 95%CI: 1.12–2.34, in EDHS-2005 vs. 3.76, 95%CI: 2.94–4.80 in EDHS-2014); and among normal compared to high risk birth weight babies (aOR: 0.79, 95%CI: 0.62–0.99 in EDHS-2005 *P* < 0.05 vs. 0.83, 95%CI: 0.65–1.04 in EDHS-2014, *P* > 0.05).

**Conclusions:**

Results showed a steady rise in c-sections in Egypt that has reached an alarming level in recent years. This increase appears to be associated with a shift towards delivery in private health care facilities. More vigilance of c-section deliveries, particularly in the private sector, is warranted.

**Electronic supplementary material:**

The online version of this article (10.1186/s12884-017-1591-2) contains supplementary material, which is available to authorized users.

## Background

Caesarean section (c-section) delivery is a major surgical operation aimed at saving lives [1]. Globally, the proportion of c-sections, one of the most common surgeries, continues to rise particularly in high- and middle-income countries [1]. Caesarean sections should be performed when vaginal delivery poses a risk to the mother or baby as in case of prolonged labor, fetal distress, or fetal mal-presentation. The World Health Organization (WHO) underscores the importance of focusing on the needs of the pregnant mothers and discourages performing c-sections with no need. Caesarean delivery without a medical need places mothers and their babies at-risk of short- and long-term health consequences [1]. It is associated with increased risk of blood transfusion, hysterectomy, maternal and child death [2–4], uterine rupture, placenta accreta, and placenta previa [5, 6]. It also costs more and requires longer hospitalization than vaginal delivery [7].

The WHO has considered a population-based rate of c-sections between 10 and 15%, as an ideal rate that was associated with a notable decline in maternal mortality ratio (MMR) and neonatal mortality rate (NMR) [1]. In 2008, half of 137 countries have exceeded this recommended threshold [8]. In 23 out of 24 countries, proportion of c-sections without medical indication ranged between 0.01% and 2% [3]. Caesarean section rate varied across different countries, worldwide. The rate was between 20% and 22.5% in the United Kingdom and Canada. In Italy and South America, c- section rate was as high as 85% [9–11]. In Saudi Arabia, the c-section rate increased by 80%, from 10.6% to 19.1%, between the years 1997 and 2006 [12]. Private practice has contributed to the increased c- section rate internationally. In Rome, the rate of c-section delivery was reported to be approximately 44%; 85% of all birth in some private clinics of Rome were c-section [13]. In Jordan, a neighboring country to Egypt, between 2002 and 2012, the c-section deliveries increased significantly over time, from 18.2% in 2002 to 30.3% in 2012; an increase by 70% in c-sections was in the private hospitals [14].

Health services in Egypt are provided by three sectors based on the financing source: the public, the parastatal, and the private sector. The public sector covers the government and quasi-governmental hospitals (parastatal). Public hospitals receive funding from the Ministry of Finance while the government ministries have a controlling share of decision making in parastatal hospitals [15]. Public hospitals include a total of 1,048 inpatient facilities with more than 80,000 beds. The private sector has a total of 2,024 inpatient facilities with a total of 22,647 beds that accounts for approximately 16% of the total inpatient bed capacity in Egypt [15].

In the past two decades, there were significant achievements in matters related to maternal health in Egypt. Home-based deliveries declined by over 60% [16], medically assisted births rose sharply from 35% in 1988 to 92% in 2014, and 90% of mothers received antenatal care (ANC) services from a trained provider [16]. The MMR declined from 174/100,000 live births in 1992 to 54/100,000 in 2010 [17]. However, over the last two years, the MMR has slightly increased to 57/100,000; 23.5% of these mortalities delivery were initiated in a private clinic [17]. The NMR was 14/1,000 births during the five-year period prior to 2014 [16].

The last study tracking changes in c-sections in Egypt was reported in 2004 [18]. According to which, based on data from two Egyptian Demographic and Health Surveys (EDHS), the institutional-based proportion of c-sections increased from 13.9% in 1988 to 22% in 2000 [18]. Birth delivery in the private sector was associated with this increase [18]. However, this study did not explore the change in c-section rate among mothers who are potentially at low risk for c-section. Although EDHS is a non-institutional-based survey, it is reported that the DHS data on c-section deliveries are sufficiently reliable for national and global monitoring purposes since the recall bias on reporting a major surgical procedure is very low [19]. With the increased access to healthcare services in Egypt, represented by a decline in home-based deliveries and an increase in ANC services [16], the specific objectives of this study are: to (1) assess the trend of c-section deliveries and (2) to identify factors associated with the over time change in c-sections in Egypt, from 2005 to 2014, with a focus on the role of place of delivery in performing c-sections, particularly for mothers who are potentially at low risk for c-section delivery.

## Methods

### Data sources

The EDHS survey aimed at providing national estimates with special emphasis on maternal and child health [16]. Employing a standardized and rigorous sampling and data collection methodology, the survey collected information from a nationally representative sample of Egyptian individuals with a large sample size [16]. Details related to sampling design, sample size, study instruments, data collection, how informed consent was obtained, and other related methodology are described elsewhere [16]. The EDHS data are accessible from the Measure DHS website [20]. The STROBE statement for reporting observational studies was followed [21] (STROBE checklist can be found in the Additional file 1).

### Study population

**Fig. 1:**
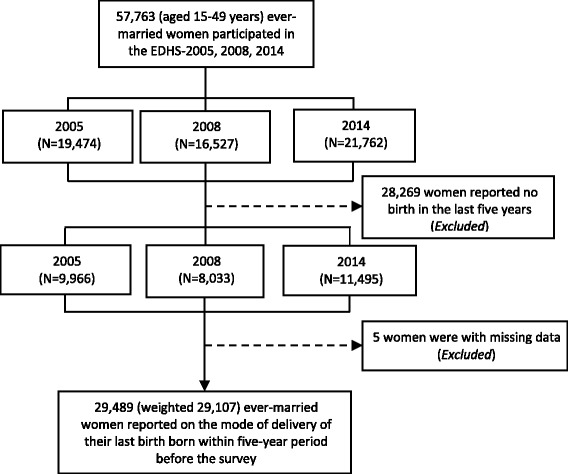
Sample selection, unweighted numbers

For the purpose of this study, the 2005, 2008, and 2014 EDHS ever-married women databases were merged based on established guidelines for managing DHS data [22]. All mothers who replied by “yes” or “no” to the question “*Has your last baby born in the past five years, including this year, was delivered by a c-section or normal/vaginal delivery?*” were included in the analysis, leaving a final sample of 29,489 mothers (weighted sample of 29,107) after excluding five women with missing data and 28,269 women who did not report giving birth within the prior five years (Fig. 1).

### Study variables

#### Sociodemographics

Specific information collected in the EDHS that reported [14, 18, 23] or could potentially have an influence on the mode of birth delivery was characterized. Sociodemographic characteristics included mother’s age at last birth (≤18, 19–24, 25–29, 30–34, and ≥35 years). Mother’s socioeconomic status (SES) was defined based on composite scores of the mother’s education level and household’s wealth status. This was performed to address the problem of multicollinearity between education and income [24]. First, mothers were dichotomized according to their education level as either with “low education” (primary or below) or with “high education” (secondary or above) and trichotomized them according to their wealth status into tertiles: poor, middle, or rich. Details related to the EDHS wealth index can be found elsewhere [16]. Then, based on the sum of scores of education and wealth variables, mothers with low SES (poor and with low education), middle SES (either poor or with low education), and high SES (non-poor and with high education) were identified [24].

Mothers were categorized according to their lifetime parity into primigravida, multipara with 2–3, or multipara with ≥4 births. Female genital mutilation (FGM) status was reported in a binary variable (yes or no). Body mass index (BMI) (kg/m^2^) was calculated as the ratio of the weight in kilograms divided by height squared (m^2^) data that were collected at the time of survey. Mother’s BMI was trichotomized into normal (BMI = 18.5–24.9 kg/m2), underweight (BMI <18.5 kg/m2), or overweight/obese (BMI ≥25 kg/m2) [25]. Maternal BMI has been linked to increased risk of adverse pregnancy outcomes leading to c-section delivery. Underweight and overweight/obese mothers were defined as a “high-risk” group for c-section. Underweight mothers are more likely to have underweight babies and pre-term delivery [26] while overweight/obese mothers are more likely to have overweight babies, preeclampsia, macrosomia, and gestational diabetes [27–30].

#### Spatial characteristics

Spatial characteristics included place of residence (rural or urban) and geographical region (urban governorates, urban or rural-lower Egypt, and urban or rural-upper Egypt).

#### Newborn characteristics

Mothers were categorized according to the birth weight as mother with: normal (2,500–3,999 g), low (<2,500 g), or high (≥4,000 g) birth weight baby. According to the birth multiplicity of the last birth born within the 5 years prior to the EDHS survey, mothers were categorized into two groups (singleton or multiple births). Mothers reported singleton or normal birth weight (2,500 to 3,999 g) babies were categorized as “low risk” while multiple or low/high birth weight (<2,500 g or ≥4,000 g) babies were categorized as a “high risk” group for c-section [14, 23]. Low/high birth weight or multiple births are main obstetric variables to increase risk of c-section [23, 31]. The mode of last birth delivery in relation to mode of delivery of the previous birth occurred within the five years of survey was categorized into vaginal after c-section, repeated c-section, repeated vaginal, or c-section after vaginal. Primigravida mothers were also included within this variable.

#### Institutional characteristics

According to the number of ANC visits made by mother during her last pregnancy, mothers were categorized into four groups (no visits, 1–3, ≥4, or don’t know/missing). Place of birth delivery was categorized into: home, public sector including parastatal health care facilities, or private sector.

### Statistical analyses

Frequency of mothers for each survey and their percentage distribution per each measured characteristic for all births and for institutional-based births were calculated, by survey round. Also, the proportion of institutional-based c-sections for each measured characteristic by survey round were recorded and assessed the over time trend of c-sections for each measured category’s item by chi-square tests for trend.

The crude and adjusted trend of c-sections utilizing the EDHS-2005 as a reference in binary and multivariable logistic regression models for all institutional-based deliveries, and stratified by place of delivery, after merging the datasets were subsequently investigated. This stratification was done to examine the role of place of delivery on the over time trend of c-sections in Egypt. In the binary logistic regression models, *P*-value for the trend was assessed for each model by entering the year of the survey as a continuous variable.

The quantitative association between institutional-based c-sections and each measured characteristic for each survey separately was also investigated. The crude (ORs) and adjusted odds ratios (aORs) and their respective 95% confidence intervals (CIs) were reported. To adjust for any potential confounding effect, all covariates were entered under analysis simultaneously in multivariable models.

A place of delivery-stratified multivariable logistic regression analysis was performed to quantify the association between institutional-based c-sections and the measured characteristics for each survey round. This was performed to understand which factors were associated with increased trend of c-sections in Egypt, stratified by place of delivery.

The sampling weights available in the EDHS databases were applied in all of performed statistical calculations. The data were analyzed using IBM SPSS version 22.0 (IBM Corp., Armonk, NY, USA) [32]. *P*-values <0.05 were considered statistically significant.

## Results

The three EDHSs were based on a nationally representative sample of 57,763 ever-married women aged 15 to 49 years, of which, 29,107 mothers reported on the mode of delivery of their last birth born within five years preceding each survey (Fig. 1).

Table 1 presents descriptive characteristics of all mothers and mothers who reported institutional-based delivery by survey round. The table shows that the institutional-based last birth occurred at mother’s age of ≥35 years or ≤18 years declined slightly from 11.4% to 10.2% and from 4.9% to 3.7%, from EDHS-2005 to EDHS-2014, respectively. Percentage of mothers with low SES who reported institutional-based birth declined by 4.9 percentage points mainly for an increase by 3.9 percentage points for the favor of mothers with a medium SES. Percentage of primigravida or mothers with ≥4 children declined from 28.2% to 24.3% and from 21.7% to 20.7%, respectively. Percentage of overweight mothers increased from 76.2% to 79.6%. Over the study period, percentage of institutional-based normal birth weight babies increased by 13.6 percentage points. Institutional-based c-sections increased from 32.4% in EDHS-2005 to 61.8% in EDHS-2014. Repeated c-sections increased by 10 percentage points along a slight increase in c-sections after vaginal delivery by only 1.1 percentage points. Birth delivery declined in public sector by 11.8 percentage points for the favor of the private sector.

**Table 1:** Descriptive characteristics of all mothers (*N* = 29,107) and mothers with the last institutional-based (*N* = 22,194) birth occurred within five years prior to each survey, by survey round

	EDHS-2005		EDHS-2008		EDHS-2014	
	All births *N* = 9823	Institutional births *N* = 6517	All births *N* = 7893	Institutional births *N* = 5713	All births *N* = 11,391	Institutional births *N* = 9964
	%	%	%	%	%	%
*Socio-demographic*						
Age at last birth, years						
≥ 35	11.7	11.4	11.1	11.2	10.5	10.2
30–34	18.2	18.3	16.6	16.4	19.1	18.8
25–29	29.2	30.3	30.6	31.2	32.9	33.2
19–24	36.0	35.1	36.8	36.3	33.8	34.1
≤ 18	4.9	4.9	4.9	4.8	3.7	3.7
Socioeconomic status						
Low	28.4	18.7	24.6	16.6	16.7	13.8
Medium	26.2	24.5	25.1	23.5	29.6	28.4
High	45.4	56.8	50.1	60.0	53.7	57.9
Lifetime parity						
Multipara with ≥4 children	27.5	21.7	23.7	19.2	23.1	20.7
Multipara with 2–3 children	48.7	50.1	49.7	50.2	54.2	55.0
Primigravida	23.8	28.2	26.6	30.6	22.7	24.3
FGM^a^						
No	3.9	4.9	4.7	5.7	8.6	9.2
Yes	96.0	95.1	95.3	94.3	91.4	90.7
*Missing*	0.01					
BMI						
Normal 18.5–24.9 (low risk)	25.7	22.3	28.7	25.5	19.8	19.3
Underweight <18.5 (high risk)	0.6	0.5	0.6	0.4	0.3	0.3
Overweight ≥25 (high risk)	72.7	76.2	70.1	73.4	79.0	79.6
*Missing*	1.0	1.1	0.6	0.7	0.8	0.8
*Spatial*						
Residence						
Urban	38.1	48.0	38.1	45.3	31.8	34.3
Rural	61.9	52.0	61.9	54.7	68.2	65.7
Region						
Urban governorates	14.8	19.7	16.4	20.4	10.8	11.8
Urban-Lower Egypt	10.0	13.1	10.1	12.0	9.4	10.4
Rural-Lower Egypt	31.1	32.2	34.3	35.4	39.0	40.2
Urban-Upper Egypt	12.6	14.4	10.8	11.9	11.1	11.5
Rural-Upper Egypt	30.1	19.4	27.3	18.8	28.8	25.2
Frontier governorates	1.2	1.1	1.4	1.5	0.9	0.9
*Newborn characteristics*						
Birth weight						
Normal (2,500–3,999 g)	30.5	39.0	35.1	43.5	49.4	52.6
High risk (<2,500, ≥4,000 g)	8.7	10.8	8.1	10.1	13.5	14.1
*Not weighted/Missing*	60.8	50.2	56.8	46.3	37.1	33.3
Birth multiplicity						
Singleton birth	97.6	97.0	97.9	97.4	97.8	97.6
Multiple birth	2.4	3.0	2.1	2.6	2.2	2.4
Delivery mode						
Vaginal	78.5	67.6	70.8	59.7	45.9	38.2
C-section	21.5	32.4	29.2	40.3	54.1	61.8
Delivery mode in relation to the previous^b^						
Primigravida	66.0	69.0	69.0	70.5	66.2	66.8
Vaginal after caesarean	0.5	0.4	0.4	0.4	0.4	0.3
Repeated caesarean	5.2	7.8	6.9	9.5	15.6	17.8
Repeated vaginal	27.0	20.9	22.2	17.5	15.2	12.1
Caesarean after vaginal	1.3	1.9	1.5	2.1	2.6	3.0
*Institutional*						
Antenatal care visits						
No visits	28.5	18.3	25.8	17.1	9.7	7.3
1–3	10.1	8.3	6.9	6.0	7.1	6.1
≥ 4	60.7	72.8	66.5	76.2	82.8	86.2
Don’t know/missing	0.7	0.6	0.8	0.7	0.4	0.4
Place of delivery						
Home	33.6	0.0	27.6	0.0	12.5	0.0
Public sector	26.9	40.6	27.2	37.6	25.2	28.8
Private sector	39.5	59.4	45.2	62.4	62.3	71.2
*Missing*		4		3		

Weighted numbers and percentages

BMI: body mass index

^a^ female genital mutilation, ^b^ previous birth occurred within past five years

**Fig. 2:**
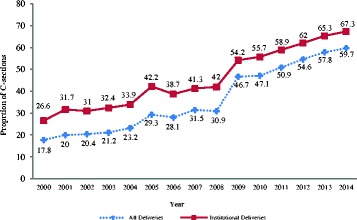
Trend in c-section deliveries in Egypt, 2000–2014

Proportion of c-sections by year showed that the population-based trend of c-sections increased exponentially from 17.8% in 2000 to 59.7% in 2014 that further increased from 26.6% to 67.3% when the analysis was limited to the institutional-based deliveries, during the same study period (Fig. 2).

Table 2 shows the over time changes in proportion of institutional-based c-sections. From EDHS-2005 to EDHS-2014, proportion of c-sections increased among mothers in each measured characteristic within each measured category. Largest increases were among mothers aged ≤18 years (34.1 points), primigravida mothers (34.4 points), mothers with normal BMI (31.4 points), normal birth weight (30.1 points) or singleton babies (29.5 points), and in the private sector (32.6 points). Of the 6158 institutional-based c-sections performed in EDHS-2014, 77.3% were performed in the private sector.

**Table 2:** Institutional-based proportion of c-section deliveries among mothers with a last birth occurred within five years prior to each survey, by survey round

	EDHS-2005 N = 6517	EDHS-2008 N = 5713	*P-value* ^a^	EDHS-2014 N = 9964	*P-value* ^*b*^	Absolute difference
	C-section, %	C-section, %		C-section, %		(% in 2014–% in 2005
*Socio-demographic*						
Age at last birth						
≥ 35	37.2	44.3	0.007	62.4	<0.001	25.2
30–34	35.5	43.1	<0.001	62.4	<0.001	26.9
25–29	33.6	42.3	<0.001	61.5	<0.001	27.9
19–24	29.2	37.1	<0.001	61.8	<0.001	32.6
≤ 18	25.8	33.6	0.038	59.9	<0.001	34.1
*P-value* ^*b*^	<0.001	<0.001		0.903		
Socioeconomic status						
Low	22.7	33.5	<0.001	51.6	<0.001	28.9
Medium	29.2	35.2	0.001	57.9	<0.001	28.7
High	37.0	44.2	<0.001	66.1	<0.001	29.1
*P-value* ^*b*^	<0.001	<0.001		<0.001		
Lifetime parity						
Multipara with ≥4 children	27.3	33.3	0.001	52.2	<0.001	24.9
Multipara with 2–3 children	32.9	40.8	<0.001	61.8	<0.001	28.9
Primigravidas	35.6	43.9	<0.001	70.0	<0.001	34.4
*P-value* ^*b*^	<0.001	<0.001		<0.001		
FGM^c^						
No	44.8	53.5	0.027	68.9	<0.001	24.1
Yes	31.8	39.5	<0.001	61.1	<0.001	29.3
*P-value* ^*b*^	<0.001	<0.001		<0.001		
*Missing*	5	0		3		
BMI						
Normal (18.5–24.9)	26.2	36.3	<0.001	57.6	<0.001	31.4
High risk (<18.5, ≥25)	34.2	41.6	<0.001	62.8	<0.001	28.6
*P-value* ^*b*^	<0.001	<0.001		<0.001		
*Missing*	69	41		80		
*Spatial*						
Residence						
Urban	36.8	44.5	<0.001	65.8	<0.001	29.0
Rural	28.4	36.9	<0.001	59.7	<0.001	31.3
*P-value* ^*b*^	<0.001	<0.001		<0.001		
Region						
Urban governorates	40.4	44.6	0.034	67.3	<0.001	26.9
Urban-Lower Egypt	42.0	48.8	0.008	73.6	<0.001	31.6
Rural-Lower Egypt	32.3	38.4	<0.001	65.8	<0.001	33.5
Urban-Upper Egypt	27.6	41.2	<0.001	57.8	<0.001	30.2
Rural-Upper Egypt	22.2	34.4	<0.001	50.2	<0.001	28.0
Frontier governorates	24.7	28.9	0.550	51.6	0.002	26.9
*P-value* ^*b*^	<0.001	<0.001		<0.001		
*Newborn characteristics*						
Birth weight						
Normal (2,500–3,999)	35.1	41.4	<0.001	65.2	<0.001	30.1
High risk (<2,500, ≥4,000)	41.6	51.1	0.001	67.1	<0.001	25.5
*P-value* ^*b*^	0.002	<0.001		0.182		
*Not weighted/Missing*	3272	2648		3317		
Birth multiplicity						
Singleton birth	32.0	40.1	<0.001	61.5	<0.001	29.5
Multiple birth	48.0	50.0	0.709	75.2	<0.001	27.2
*P-value* ^*b*^	<0.001	0.016		<0.001		
Mode of delivery in relation to the previous birth^d^						
Primigravidas mother	32.9	40.8	<0.001	61.4	<0.001	28.5
Vaginal after caesarean	–	–	–	–	–	
Repeated caesarean	–	–	–	–	–	
Repeated vaginal	–	–	–	–	–	
C-section after vaginal	–	–	–	–	–	
*Institutional*						
Antenatal care visits						
No visits	18.8	30.1	<0.001	42.4	<0.001	23.6
1–3	26.7	34.1	0.019	48.6	<0.001	21.9
≥ 4	36.4	43.3	<0.001	64.4	<0.001	28.0
*P-value* ^*b*^	<0.001	<0.001		<0.001		
*Don’t know/missing*	40	41		36		
Place of delivery						
Public sector	29.4	35.4	<0.001	48.5	<0.001	19.1
Private sector	34.4	43.2	<0.001	67.0	<0.001	32.6
*P-value* ^*b*^	<0.001	<0.001		<0.001		

^a^ *P*-value, assessed over time differences in proportion of institutional-based caesarean delivery between EDHS-2005 and EDHS-2008, and between EDHS-2008 and EDHS-2014

^b^ *P*-value, assessed differences in proportion of institutional-based caesarean delivery between each measured sub-categories in each survey

^c^ female genital mutilation

^d^ previous birth occurred within the past five years

The multivariable analysis revealed a 3.46-time (95% CI: 3.15–3.80) increase in trend of c-sections in EDHS-2014 relative to EDHS-2005. The further place of delivery-stratified multivariate analysis showed that this increase in trend of c-sections was higher in private sector (aOR, 4.19, 95% CI: 3.73–4.70) than in public sector (aOR, 2.67, 95% CI: 2.27–3.13) (Table 3).

**Table 3:** Bivariate and multivariate logistic regression for the trend of institutional-based c-section delivery among all mothers, stratified by place of delivery

Among all mothers	OR (95% CI)		aOR (95% CI)	
Survey round (Ref: EDHS-2005)				
EDHS-2008	1.41 (1.31–1.52)^***^		1.39 (1.25–1.54)^***^	
EDHS-2014	3.37 (3.20–3.60)^***^		3.46 (3.15–3.80)^***^	
Place of delivery	Public sector		Private sector	
Survey round (Ref: EDHS-2005)	OR (95% CI)	aOR (95% CI)	OR (95% CI)	aOR (95% CI)
EDHS-2008	1.32 (1.16–1.49)^***^	1.35 (1.13–1.61)**	1.45 (1.33–1.60)^***^	1.42 (1.25–1.62)^***^
EDHS-2014	2.26 (2.02–2.53)^***^	2.67 (2.27–3.13)**	2.88 (3.58–4.21)^***^	4.19 (3.73–4.70)^***^

OR: odds ratio, aOR: adjusted odds ratio (for all covariates under analysis), CI: confidence interval

*P*-value for the trend was assessed by entering the year of the survey as a continuous variable

^*^ *P* < 0.05, ^**^ *P* = 0.001, ^***^ *P* < 0.001

Table 4 shows the results of bivariate and multivariable logistic regression of association between institutional-based c-section and measured characteristics by survey round. As the table shows, relative to mothers aged ≥35 years, in EDHS-2014, the trend of c-section among mothers aged ≤18 years (aOR, 0.33, 95% CI: 0.23–0.47) or 19–25 years (aOR, 0.41, 95% CI: 0.31–0.55) was higher when it compared with c-sections in EDHS-2005 (aOR, 0.26, 95% CI: 0.16–0.50 and 0.31, 95% CI: 0.22–0.42, respectively). In EDHS-2005, mothers from high SES (aOR, 1,78, 95% CI: 1.25–2.54, *P* = 0.001) were more likely to undergo c-section relative to those with low SES, but in EDHS-2014 this observed significant difference disappeared. Among primigravida, trend of c-sections in EDHS-2014 (aOR, 5.57, 95% CI: 4.46–6.97) was higher than that in EDHS-2005 (aOR, 2.91, 95% CI: 2.11–4.00). Normal birth weight babies had higher odds undergoing c-section delivery in EDHS-2014 (aOR, 0.86, 95% CI: 0.75–0.98) compared to EDHS-2005 (aOR, 0.76, 95% CI: 0.63–0.91).

**Table 4:** Bivariate and multivariate logistic regression for the association between institutional-based c-section as dependent variable and measured characteristics by the survey round

Characteristics	EDHS-2005		EDHS-2008		EDHS-2014	
	OR (95% CI)	aOR (95% CI)	OR (95% CI)	aOR (95% CI)	OR (95% CI)	aOR (95% CI)
*Socio-demographic*						
Age at last birth (Ref: ≥35 yrs)						
30–34	0.93 (0.77–1.12)	0.77 (0.57–1.04)	0.93 (0.77–1.12)	0.83 (0.61–1.11)	1.00 (0.86–1.17)	0.77 (0.61–0.95)^*^
25–29	0.85 (0.71–1.01)	0.47 (0.35–0.65)^***^	0.85 (0.71–1.01)	0.72 (0.55–0.95)^**^	0.97 (0.84–1.11)	0.54 (0.44–0.66)^***^
19–24	0.70 (0.59–0.83)^***^	0.31 (0.22–0.42)^***^	0.70 (0.59–0.83)^***^	0.41 (0.31–0.55)^***^	0.98 (0.85–1.13)	0.41 (0.31–0.55)^***^
≤ 18	0.58 (0.44–0.78)^***^	0.26 (0.16–0.50)^***^	0.63 (0.47–0.85)^***^	0.37 (0.23–0.60)^***^	0.90 (0.71–1.15)	0.33 (0.23–0.47)^***^
Socioeconomic status (Ref: low)						
Medium	1.40 (1.18–1.67)^***^	1.55 (1.07–2.24)^*^	1.08 (0.91–1.29)	0.79 (0.58–1.07)	1.29 (1.13–1.47)^***^	0.98 (0.80–1.21)
High	2.0 (1.72–2.32)^***^	1.78 (1.25–2.54)^**^	1.57 (1.35–1.83)^***^	0.77 (0.57–1.04)	1.83 (1.63–2.07)^***^	0.97 (0.85–1.12)
Lifetime parity (Ref: multipara with ≥4 children)						
Multipara with 2–3 children	1.31 (1.14–1.50)^***^	1.06 (0.81–1.38)	1.38 (1.19–1.59)^***^	1.41 (1.08–1.84)^*^	2.13 (1.89–2.41)^***^	1.52 (1.27–1.81)^***^
Primigravidas	1.47 (1.23–1.71)^***^	2.91 (2.11–4.00)^***^	1.57 (1.34–1.83)^***^	3.88 (2.83–5.33)^***^	1.48 (1.34–1.64)^***^	5.57 (4.46–6.97)^***^
FGM^a^ (Ref: no)						
Yes	0.57 (0.46–0.72)^***^	0.69 (0.51–0.94)^*^	0.57 (0.45–0.71)^***^	0.61 (0.44–0.84)^**^	0.71 (0.61–0.82)^***^	0.93 (0.76–1.13)
BMI (Ref: normal 18.5–24.9)						
High risk (<18.5, ≥25)	1.46 (1.28–1.67)^***^	1.27 (1.02–1.59)^*^	1.26 (1.11–1.42)^***^	1.38 (1.13–1.77)^**^	1.25 (1.13–1.38)^***^	1.44 (1.24–1.69)^***^
*Spatial*						
Residence (Ref: urban)						
Rural	0.68 (0.61–0.76)^***^	0.78 (0.09–6.56)	0.73 (0.66–0.81)^***^	0.66 (0.12–3.52)	0.77 (0.71–0.84)^***^	0.73 (0.19–2.68)
Region (Ref: urban governorates)						
Urban-Lower Egypt	2.07 (1.20–3.56)^**^	0.94 (0.73–1.21)	1.93 (1.19–3.15)^**^	1.08 (0.81–1.43)	1.93 (1.26–2.96)^**^	1.16 (0.91–1.47)
Rural-Lower Egypt	2.21 (1.27–3.83)^**^	0.99 (0.12–8.35)	2.29 (1.39–3.75)^**^	1.40 (0.26–7.61)	2.62 (1.70–4.04)^***^	1.08 (0.29–4.00)
Urban-Upper Egypt	1.45 (0.85–2.49)	0.50 (0.38–0.67)	1.49 (0.92–2.41)	0.90 (0.68–1.17)	1.81 (1.19–2.74)^**^	0.62 (0.49–0.77)^***^
Rural-Upper Egypt	1.17 (0.67–2.03)	0.90 (0.10–7.66)	1.68 (1.02–2.76)^*^	1.27 (0.23–6.92)	1.28 (0.84–1.97)	0.74 (0.20–2.75)
Frontier governorates	0.87 (0.51–1.51)	0.47 (0.18–1.22)	1.26 (0.77–2.05)	0.56 (0.25–1.25)	0.95 (0.62–1.44)	0.69 (0.33–2.74)
*Newborn characteristics*						
Birth weight (Ref: high risk <2,500, ≥4,000)						
Normal 2,500–3,999	0.75 (0.64–0.90)^**^	0.76 (0.63–0.91)^**^	0.67 (0.56–0.81)^***^	0.65 (0.54–0.79)^***^	0.92 (0.81–1.04)	0.86 (0.75–0.98)^*^
Birth multiplicity (Ref: multiple)						
Singleton	0.51 (0.38–0.68)^***^	0.44 (0.30–0.65)^**^	0.67 (0.48–0.92)^*^	0.50 (0.32–0.781)^**^	0.52 (0.39–0.70)^***^	0.46 (0.32–0.66)^***^
Previous birth c-section^b^ (Ref: no)						
Yes	66.3 (44.3–99.2)^***^	113 (58.4–218)^***^	61.4 (39.6–95.1)^***^	114.4 (55.3–236)^***^	69.4 (47.9–100)^***^	123.3 (71.9–211)^***^
Primigravidas	1.87 (1.64–2.10)^***^	2.76 (2.23–3.41)^***^	1.90 (47.9–100)^***^	2.83 (2.30–3.49)^***^	2.64 (2.38–2.91)^***^	3.82 (3.27–4.46)^***^
*Institutional*						
ANC (Ref: no visits)						
1–3 visits	1.56 (1.24–2.00)^***^	1.58 (1.01–2.47)^**^	1.20 (0.93–1.56)	1.18 (0.76–1.85)	1.28 (1.04–1.60)^*^	0.87 (0.61–1.24)
≥ 4 visits	2.48 (2.11–2.90)^***^	1.64 (1.21–2.23)^**^	1.77 (1.52–2.06)^***^	1.51 (1.17–1.94)^**^	2.45 (2.10–2.90)^***^	1.31 (1.01–1.70)^*^
Place of delivery (Ref: public)						
Private sector	1.26 (1.13–1.40)^***^	1.36 (1.14–1.62)^**^	1.39 (1.26–1.55)^***^	1.40 (1.17–1.66)^***^	2.16 (1.98–2.36)^***^	1.96 (1.72–2.23)^***^

Number of cases included in multivariate models was only 12,894 (9236 children were not weighted and 64 mothers did not report on the ANC visits)

OR: odds ratio, aOR: adjusted odds ratio (for all covariates under analysis), CI: confidence interval, ANC: antenatal care

^a^ female genital mutilation, ^b^ previous birth occurred within the past five years

^*^ *P* < 0.05, ^**^ *P* = 0.001, ^***^ *P* < 0.001

Furthermore, place of delivery-stratified multivariable logistic regression demonstrated that in private sector, mothers aged ≤18 years had less likelihood to undergo c-sections in EDHS-2005 (aOR, 0.28, 95% CI: 0.15–0.53) compared to EDHS-2014 (aOR, 0.40, 95% CI: 0.26–0.63). However, in EDHS-2014 same age group of mothers was 84% less likely to undergo c-sections in the public sector. In public sector, SES was not associated with c-section delivery in all survey rounds, but in private sector, the significant association of SES with c-section observed in EDHS-2005 disappeared in EDHS-2014. The likelihood of primigravida mothers to undergo c-section in public sector declined from 2.21-time (95% CI: 1.37–3.58) to 1.72-time (95% CI: 1.18–2.50) whereas it increased in private sector from 1.62-times (95% CI: 1.12–2.34) to 3.76-times (95% CI: 2.94–4.80), in EDHS-2005 to EDHS-2014, respectively. Normal birth weight babies were significantly at lower risk to undergo c-section in EDHS-2005, but in EDHS-2014 they had an equal likelihood with high-risk birth weight babies in both public and private sectors (Table 5).

**Table 5:** Place of delivery-stratified multivariate logistic regression for the association between institutional-based c-section delivery as dependent variable and measured characteristics, by the survey round

	EDHS-2005		EDHS-2008		EDHS-2014	
	Public	Private	Public	Private	Public	Private
	aOR (95% CI)		aOR (95% CI)		aOR (95% CI)	
*Socio-demographic*						
Age at last birth (Ref: ≥35 yrs)						
30–34	0.23 (0.09–0.62)^**^	0.59 (0.41–0.87)^**^	0.70 (0.43–1.15)	0.93 (0.63–1.36)	0.75 (0.51–1.10)	0.79 (0.61–1.07)
25–29	0.33 (0.19–0.54)^***^	0.40 (0.28–0.56)^***^	0.89 (0.57–1.40)	0.66 (0.46–0.93)^*^	0.52 (0.36–0.74)^***^	0.56 (0.44–0.72)^***^
19–24	0.71 (0.44–1.14)	0.31 (0.21–0.45)^***^	0.37 (0.23–0.61)^***^	0.44 (0.30–0.64)^***^	0.39 (0.27–0.57)^***^	0.43 (0.33–0.56)^***^
≤ 18	1.23 (0.77–1.98)	0.28 (0.15–0.53)^***^	0.27 (0.11–0.67)^**^	0.43 (0.24–0.79)^**^	0.16 (0.07–0.35)^***^	0.40 (0.26–0.63)^***^
Socioeconomic status (Ref: low)						
Medium	1.31 (0.76–2.27)	1.71 (1.04–2.83)^*^	1.23 (0.74–2.06)	0.59 (0.39–0.88)^**^	0.86 (0.60–1.22)	1.07 (0.83–1.38)
High	1.51 (0.90–2.55)	2.04 (1.26–3.29)^**^	1.25 (0.76–2.05)	0.61 (0.42–0.88)^**^	1.11 (0.78–1.59)	0.97 (0.76–1.25)
Lifetime parity (Ref: ≥4)						
2–3	1.54 (1.05–2.26)^*^	1.04 (0.76–1.41)	1.41 (0.95–2.07)	2.20 (1.60–3.03)^***^	1.41 (1.05–1.89)^*^	1.82 (1.51–2.21)^***^
Primigravidas	2.21 (1.37–3.58)^**^	1.62 (1.12–2.34)^*^	1.50 (0.92–2.44)	3.73 (2.56–5.42)^***^	1.72 (1.18–2.50)^**^	3.76 (2.94–4.80)^***^
FGM^a^ (Ref: no)						
Yes	0.77 (0.41–1.45)	0.68 (0.48–0.97)^*^	0.51 (0.24–1.06)	0.62 (0.43–0.89)^*^	0.81 (0.55–1.20)	0.95 (0.75–1.20)
BMI (Ref: normal 18.5–24.9)						
High risk (<18.5, ≥25)	1.14 (0.78–1.67)	1.34 (1.01–1.77)^*^	1.24 (0.88–1.76)	1.52 (1.19–1.92)^**^	1.78 (1.32–2.40)^***^	1.33 (1.11–1.60)^**^
*Spatial*						
Residence (Ref: urban)						
Rural	0.82 (0.60–10.9)	0.70 (0.01–36.73)	0.95 (0.10–8.69)	0.52 (0.04–7.05)	0.56 (0.08–4.04)	1.0 (0.14–6.96)
Region (Ref: urban governorates)						
Urban-Lower Egypt	1.14 (0.73–1.79)	0.91 (0.66–1.24)	0.95 (0.57–1.62)	1.13 (0.79–1.60)	1.04 (0.69–1.56)	1.19 (0.88–1.60)
Rural-Lower Egypt	1.06 (0.08–14.7)	1.07 (0.02–56.6)	1.29 (0.14–12.19)	1.56 (0.11–21.4)	1.55 (0.21–11.4)	0.74 (0.11–5.14)
Urban-Upper Egypt	0.34 (0.19–0.58)^***^	0.61 (0.43–0.86)^**^	0.96 (0.59–1.54)	0.89 (0.64–1.24)	0.62 (0.42–0.91)^*^	0.60 (0.45–0.80)^**^
Rural-Upper Egypt	0.67 (0.05–9.46)	1.18 (0.02–63.2)	1.27 (0.14–11.9)	1.32 (0.09–18.2)	1.04 (0.14–7.67)	0.51 (0.07–3.54)
Frontier governorates	0.81 (0.24–2.71)	0.25 (0.05–1.23)	0.56 (0.18–1.77)	0.62 (0.19–1.91)	0.72 (0.19–2.75)	0.68 (0.27–1.73)
*Newborn characteristics*						
Birth weight (Ref: high risk <2,500, ≥4,000)						
Normal (2,500–3,999)	0.63 (0.47–0.84)^**^	0.79 (0.62–0.99)^*^	0.46 (0.34–0.62)^***^	0.81 (0.64–1.04)	0.83 (0.66–1.05)	0.83 (0.65–1.04)
Birth multiplicity (Ref: multiple)						
Singleton	0.39 (0.20–0.75)^**^	0.46 (0.28–0.77)^**^	1.0 (0.47–0.2.11)	0.33 (0.18–0.60)^***^	0.57 (0.32–1.03)	0.41 (0.26–0.66)^***^
Previous birth C-section^b^ (Ref: no)						
Yes	53.6 (25.8–120.4)^***^	263.3 (82.2–843)^***^	66.5 (25.1–176)^***^	236.5 (73.1–765)^***^	95.4 (41.7–218)^***^	145.3 (71.0–297)^***^
Primigravidas	2.61 (1.81–3.70)^***^	2.85 (2.19–3.72)^***^	1.84 (1.27–2.70)^**^	3.56 (2.76–4.60)^***^	2.48 (1.86–3.32)^***^	4.52 (3.75–5.45)^***^
*Institutional*						
ANC (Ref: no visits)						
1–3	0.92 (0.46–1.84)	2.33 (1.27–4.28)^**^	0.72 (0.30–1.74)	1.33 (0.77–2.29)	1.04 (0.58–1.84)	0.78 (0.49–1.24)
≥ 4	1.59 (1.03–2.44)^*^	1.71 (1.11–2.63)^*^	1.89 (1.29–2.77)^**^	1.29 (0.91–1.81)	1.44 (0.98–2.10)	1.21 (0.84–1.74)

Number of cases included in multivariate models was only 12,894 (9236 children were not weighted and 64 mothers did not report on the ANC visits)

OR: odds ratio^,^ aOR: adjusted odds ratio (for all covariates under analysis), CI: confidence interval, ANC: antenatal care

^a^ female genital mutilation, ^b^ previous birth occurred within the past five years

^*^ *P* < 0.05, ^**^ *P* = 0.001, ^***^ *P* < 0.001

## Discussion

The study estimated trend of c-sections among Egyptian mothers sampled in 2005, 2008, and 2014. There was a substantial rise in trend of institutional-based c-sections by more than three-fold, over the study period. The private sector appears to be the driver of the rising c-sections in Egypt, a substantial increase was also observed in use of this surgical procedure in public sector. The more than 4-folds increase in c-sections in the private sector was driven by substantial increases in c-sections among mothers who are potentially at low risk for c-section delivery.

In Egypt, the nearly 60% population-based proportion of c-sections performed in 2014 greatly exceeds the threshold of 10–15% recommended by WHO [1]. A population-based proportion of c-sections >10% did not lead to health improvements for mother or newborn [33]. Although the observed over time increase in c-section rate in Egypt is in line with what has been noted in many national and international studies [12, 14, 18, 34], this over time increase places Egypt as a country with the highest c-sections performed worldwide, after Brazil (45.9%) [8]. The institutional-based proportion (67.3%) of c-sections recorded in Egypt in 2014 is 2.2-time and 2.7-time higher than that recently recorded in Jordan (30.3%) [14] and in Saudi Arabia (25%) [35], respectively. The decline in home-based deliveries by over 60% merely reflects an improvement in provided health care services in Egypt. Over the past decade, per capita total expenditure on health increased from US$75.8 in 2000 to US$123.2 in 2010 [36]. However, improving administered health care services should not justify the massive increase in c-sections. This exponential rise in c-sections indicates an overuse for this surgical procedure that might be due to many c-sections may increasingly be performed without any medical indication.

This rise in c-sections would pose further economic burden in a resource limited-setting such as Egypt, which is already burdened with different economic difficulties where 26.3% of Egyptians live below the poverty line [37]. In 2008, the WHO estimated that 253,890 unnecessary c-sections had been performed with a total cost of US$ 41,085,585 per year [38]. Referring to the obtained results, the discernible increase in c-sections in Egypt in 2014, this study assumes that the unnecessary c-sections and its associated spending at least would double the ones estimated in 2008 [38]. Furthermore, the increased c-sections would pose further unfavorable health outcomes as a result of adverse outcomes associated with c-sections [5, 6] in a country already burdened with a relatively high MMR and NMR in addition to other infectious diseases mainly hepatitis C virus that infect nearly 15% of the 15 to 59 years old Egyptian people [39]. This disease alone consumes about 20% of the Ministry of Health and Population total annual budget to treat infected individuals [40]. Rigorous institutional-based study is needed to assess the impact of this high proportion of c-sections and identify the exact medical and non-medical needs for c-section deliveries for future planning and effective policy interventions.

In the three surveys, childbearing at ≤18 years or ≥35 years, living in high SES, maternal overweight/obesity, pregnancy with high-risk birth weight or multiple babies, delivery in a private sector were found as significant factors associated with c-section delivery in Egypt except for SES in EDHS-2014. Older mothers are more likely to experience different complications during pregnancy and delivery [41–45], even in the absence of complications, and they are more inclined to have c-section, especially primigravida mothers [44]. Younger mothers are more likely to have small pelvis to deliver a fetus which necessitate a c-section [46]. Women with high SES are more likely to be educated and to have higher income who tend to delay giving birth until older age, therefore, increasing their likelihood of a c-section delivery [42]. However, the disappeared association of SES with c-sections observed in EDHS-2014 merely explains the penetration of health care services to the socially and economically disadvantaged mothers, nevertheless this should not justify the increased c-sections among this socially deprived group. Maternal overweight/obesity increases the risk of different c-section inducing factors such as preeclampsia and gestational diabetes [47].

One of the principal objectives of this investigation was to identify main drivers of the increased c-sections in Egypt. Study findings documented that place of delivery is a major contributor to the exponentially increased c-sections in Egypt. Although the private sector occupies only 16% of total hospitals beds in Egypt [15], the trend of c-sections increased by more than 4-time over the study period. This finding is still persistent in Egypt and in line with the previous study reported in 2004 and analyzed comparably collected data [18]. Lack of compliance with regulations by private practitioners and inadequate enforcement of the law, public’s perception that medical services in private sector due to the availability of necessary medical technology and better inpatient quality care services for this surgical intervention, particularly in presence of near birth complications, as well as obstetricians’ predisposition to manage their time, are suggested [18, 23] and documented factors associated with the increasing c-sections in the private sector [31, 43, 48].

The study found that this sizeable rise in proportion of c-sections in the private sector was driven by increasing c-sections among mothers who are theoretically and empirically known as not at risk of c-section including mothers who fallen in an age group of 25–29 years, mothers of normal BMI, and mothers reported normal birth weight or singleton babies. This increase contributed substantially to the overall increase in c-sections in Egypt which supports the notion that a sizeable proportion of performed c-sections might be performed unnecessarily. This is in line with what has been recently reported from nationally-representative data in Jordan [14]. According to which, the rising in c-sections from 18.2% in 2002 to 30.3% in 2012, driven primarily by substantial rises among apparently low-risk mothers; mothers with normal birth weight or singleton babies [14].

It is worth to be mentioned that encouragement of vaginal delivery is very important to curb the steady rise in c-sections in Egypt. This study observed a decline by 12.8% in repeated institutional-based vaginal deliveries among mothers reported two births in five years preceded the survey. External cephalic version (turning the fetus from a breech or transverse position into a vertex position), vaginal birth after a previous c-section, and one-on-one trained support during labor were effective psychosocial and structural strategies at reducing the likelihood of c-sections, even among those who may have a medical indication [49]. Moreover, initiatives to raise peoples’ and health professionals’ awareness about the adverse outcomes associated with c-section and advantages of vaginal delivery are also urgently needed. Educating mothers about risks associated with c-section, midwifery training, and establishment of birthing centers, could also help encourage mothers to deliver vaginally.

The strengths of this study are that the data were from large, randomly selected population-based three datasets collected by accredited and reliable official entities using comparable methodology. The larger sample size and high response rates provided in the study with good statistical power and objective outcome measures. However, in population-based survey, the probability of recall bias is low since mothers who had a c-section delivery would not easily forget the mode of delivery given its surgical nature, particularly for the last birth [19]. Estimates associated with c-section were adjusted for any potential confounding effect of the measured characteristics. Stratification according to the place of delivery provided more insights about the substantial contribution of the private sector to the rising c-sections in Egypt with revealing contributing factors for the increased c-sections in this sector.

Findings from this study should be interpreted in light of the following limitations. The cross-sectional design of the EDHS limits the causality pathway with regard to the factors found associated with increased c-sections. Given the nature of the household-based survey where medical records are usually unavailable, collected data did not include information about whether the c-sections were performed under medical indications such as fetal mal-presentation or based solely on maternal demand, except for the birth weight and birth multiplicity that served as the only obstetric indicators could potentially at medically necessary c-section. Despite of these limitations, this study provided evidence-based estimates on trend of c-section deliveries in Egypt and associated factors to fine-tune strategies necessary to halt the rising c-sections in Egypt.

## Conclusions

Obtained results demonstrated that the proportion of c-sections in Egypt has been increasing steadily in recent years and has reached an alarming level. The proportion of c-sections documented in the last EDHS conducted in 2014 quadrupled the maximum threshold recommended by the WHO. The increase in number of birth deliveries occurred in the private sector appears to be associated with a shift towards delivery in private facilities. This increase in the private sector, particularly among mothers who were potentially at low risk of c-sections requires an urgent need to adopt critical policies and strategies that able to halt the steady rise in c-sections in Egypt and improve reproductive health and mothers and babies health outcomes. In the meantime, an in-depth institutional-based study collecting data on the exact indications associated with c-sections in Egypt is also necessary.

## Additional file

Additional file 1: STROBE checklist. STROBE statement. A checklist statement summarizing reporting of observational studies. (DOC 130 kb)

## Abbreviations

ANC: Antenatal care
aOR: Adjusted odds ratio
CI: Confidence interval
C-section: Caesarean section
DHS: Demographic and health surveys
EDHS: Egypt Demographic and Health Surveys
MMR: Maternal mortality ratio
NMR: Neonatal mortality rate
OR: Odds ratio
SES: Socioeconomic status
STROBE: Strengthening the Reporting of Observational Studies in Epidemiology
WHO: World Health Organization
