# Supplementary material for: Trend of caesarean deliveries in Egypt and its associated factors: evidence from national surveys, 2005–2014
Source: BMC Pregnancy Childbirth. 2017 Dec 13;17:417. doi: 10.1186/s12884-017-1591-2 (PMC5729511; doi:10.1186/s12884-017-1591-2)
Supplement: Additional file 1: — STROBE checklist. STROBE statement. A checklist statement summarizing reporting of observational studies. (DOC 130 kb) [file 12884_2017_1591_MOESM1_ESM.doc]

# Additional file

# Trend of caesarean deliveries in Egypt and its associated factors: evidence from national surveys, 2005-2014

Rami H. Al-Rifai^1,*^

^1^ Institute of Public Health, College of Medicine and Health Sciences, United Arab Emirates University, Al-Ain, United Arab Emirates.
​

*Reprints or correspondence

Rami H. Al-Rifai, PhD. Institute of Public Health, College of Medicine and Health Sciences, United Arab Emirates University, Al Ain, United Arab Emirates, P.O. Box 15551, Telephone: +(971) 3713-7450. Fax: +(97) 3767-2022. E-mail: rrifai@uaeu.ac.ae

**STROBE Statement**

**Type of study**: Population-based cross-sectional

# Title: “*Trend of caesarean deliveries in Egypt and its associated factors: evidence from national surveys, 2005-2014*”.

| The STROBE statement—Checklist of Items That Should be Addressed in Reports of Observational Studies | | |  | |
| --- | --- | --- | --- | --- |
|  | Item No | Recommendation |  | |
| **Title and abstract** | 1 | (*a*) Indicate the study’s design with a commonly used term in the title or the abstract.  The cross-sectional design of the Egypt demographic and health survey (EDHS) upon a nationally representative multi-stage sample of Egyptian women aged 15-49 years months was covered in the abstract. |  | |
|  |  | 1. Provide in the abstract an informative and balanced summary of what was done and what was found  - *Objectives*: To assess the, trend of and changes in factors associated with, a rise in c-section deliveries among the Egyptian mothers, from 2005 to 2014, by place of delivery - *Design*: Retrospective analysis of the data from three representative cross-sectional cohorts of Egyptian mothers. - *Setting*: The analyzed three EDHS reported on the mode of delivery for the last birth occurred within five years preceding each survey including place of delivery, social, and demographic information for a total sample of over 29,000 mothers in the three surveys. - *Outcomes***:** Primary outcome was the caesarean section mode of delivery for the last birth occurred within five years preceding each survey - *Results*: Population-based c-sections increased by 41.9 points from EDHS-2005 to EDHS-2014 (17.8% to 59.7%, respectively, *P_trend_*<0.001). Institutional-based c-sections increased by 40.7 points from EDHS-2005 to EDHS-2014 (aOR, 3.46, *P_trend_*<0.001). Compared to mothers with low socioeconomic status (SES), mothers with high SES had higher odds (aOR, 1.78, *P*=0.001) for c-section, but only in EDHS-2005. The adjusted trend of c-sections was found to be 4.19-times (*P<*0.001) higher in private sector while that in public sector it was 2.67-times higher, in EDHS-2014 relative to EDHS-2005. This increase is explained by significant increases among; mothers aged ≤18 years vs. ≥35 years (aOR: 0.28 in EDHS-2005 vs. 0.40 in EDHS-2014, *P*>0.0011); mothers from low SES vs. high SES strata; primigravida mothers vs. mothers with ≥4 children (aOR: 1.62 in EDHS-2005 vs. 3.76 in EDHS-2014); and among normal compared to high risk birth weight babies (aOR: 0.79 in EDHS-2005 *P*<0.05 vs. 0.86 in EDHS-2014, *P*>0.05). - *Conclusion*: The steady rise in c-sections in Egypt that has reached an alarming level in recent years. This increase appears to be associated with a shift towards delivery in private health care facilities. More vigilance of c-section deliveries, particularly in the private sector, is warranted. - *Limitations:* The cross-sectional design of the EDHS limits the causality pathway with regard to the drivers associated with increased c-sections. Given the nature of the household-based survey where medical records are usually unavailable, collected data did not include information about whether the c-sections were performed under medical indications such as fetal mal-presentation or based solely on maternal demand, except for the birth weight and birth multiplicity that served as the only obstetric indicators could potentially at medically necessary c-section. Despite of these limitations, this study provided evidence-based estimates on trend of c-section deliveries in Egypt and associated factors to fine-tune strategies necessary to halt the rising c-sections in Egypt. |  | |
| Introduction | | |  |  |
| Background/rationale | 2 | Explain the scientific background and rationale for the investigation being reported  Caesarean section (c-section) delivery is a major surgical operation aimed at saving lives. Globally, the proportion of c-sections, one of the most common surgeries, continues to rise particularly in high- and middle-income countries. Caesarean sections should be performed when vaginal delivery poses a risk to the mother or baby as in case of prolonged labor, fetal distress, or fetal mal-presentation. According to the World health organization, a population-based rate of c-sections between 10-15%, as an ideal rate that was associated with a notable decline in maternal and neonatal mortality rates.  In Egypt, in the past two decades, there were significant achievements in matters related to maternal health. Medically assisted births rose sharply from 35% in 1988 to 92% in 2014, 90% of mothers received antenatal care from a trained provider, and the maternal mortality ratio (MMR) declined from 174/100,000 live births in 1992 to 54/100,000 in 2010.  However, over the last two years, the MMR has slightly increased to 57/100,000; 23.5% of these mortalities delivery were initiated in a private clinic. The neonatal mortality rate (NMR) was 14/1,000 births during the five-year period prior to 2014. Public hospitals include a total of 1,048 inpatient facilities with more than 80,000 beds. The private sector has a total of 2,024 inpatient facilities with a total of 22,647 beds that accounts for approximately 16% of the total inpatient bed capacity in Egypt. The last study tracking changes in c-sections in Egypt was reported in 2004. Updated estimates on the trend of c-section and changes in its associated factors are needed for better informed decisions and strategic planning. |  | |
| Objectives | 3 | State specific objectives, including any pre-specified hypotheses  *Hypothesis*: The study hypothesizes that there was a significant rise in trend of c-section deliveries in Egypt and that rise has a strong link to the place of delivery.  *Objectives*: To assess trend of, and to identify factors associated with the change in, c-sections in Egypt, from 2005 to 2014, with a particular focus on the role of place of delivery in performing c-sections. |  | |
| Methods | | |  | |
| Study design | 4 | Present key elements of study design early in the paper  A detailed study design was clearly provided as indicated under the methodology section. A total of 9,966, 8,033, and 11,495 women aged 15-49 years 6-59 reported on the mode of delivery of their last birth born five years prior to 2005, 2008, and 2014 EDHS, respectively. |  | |
| Setting | 5 | Describe the setting, locations, and relevant dates, including periods of recruitment, exposure, follow-up, and data collection  The EDHS is a part of the demographic and health surveys (DHS) that conducted in the country to collect data from a nationally representative sample of households. The survey aimed at providing national estimates with special emphasis on maternal and child health. Employing a standardized and rigorous sampling and data collection methodology, the survey collected information from a nationally representative sample of non-institutionalized Egyptian individuals with a large sample size.  Three data sets were analysed from the EDHS carried out in 2005, 2008, and 2014. Data were collected through face-to-face interviews carried out by trained interviewers using standardized questionnaires and methodologies. All mothers who replied by “yes” or “no” to the question “*Has your last baby born in the past five years, including this year, was delivered by a c-section or normal/vaginal delivery?*” were included in the study. |  | |
| Participants | 6 | **(*a*)** *Cross-sectional study***-**Give the eligibility criteria, and the sources and methods of selection of participants.  The subjects of this study were restricted to mothers aged 15-49 years who replied by “yes” or “no” to the question “*Has your last baby born in the past five years, including this year, was delivered by a c-section or normal/vaginal delivery?*”. |  | |
| Variables | 7 | Clearly define all outcomes, exposures, predictors, potential confounders, and effect modifiers. Give diagnostic criteria, if applicable.  Detailed definition to the measured outcome and exposure variables measured in this study were clearly provided.  The outcome variable was the c-section mode of delivery of the last birth occurred within five years preceding of each survey.  The study specifically characterized information collected in the EDHS that reported or could potentially have an influence on the mode of birth delivery. Mother’s age at last birth, socioeconomic status (SES), lifetime parity, female genital mutilation, body mass index, and number of antenatal care visits were the measured socioeconomic and demographic variables of the mothers. Place of residence and geographical were the measured spatial variables of the mothers. Birth weight and birth multiplicity were the measured characteristics of the new born. |  | |
| Data sources/ measurement | 8* | For each variable of interest, give sources of data and details of methods of assessment (measurement). Describe comparability of assessment methods if there is more than one group.  Face-to-face and confidential interviews were used to collect information on composite indicators relevant to mother’s socioeconomic characteristics. C-section delivery was assessed by a direct answer by “yes” or “no” to a question inquired mothers on the mode of delivery of their last birth born five years of each survey round. |  | |
| Bias | 9 | Describe any efforts to address potential sources of bias  To avoid reporting and measurement bias, subjects were interviewed confidentially and anonymously. In the three EDHS surveys, a comparable and standardized data collection instruments and methodology was implemented. |  | |
| Study size | 10 | Explain how the study size was arrived at.  Using multi-stage sampling approach, the study subjects were representative of the all governorates in Egypt. |  | |
| Quantitative variables | 11 | Explain how quantitative variables were handled in the analyses. If applicable, describe which groupings were chosen and why.  Quantitative variables were reported as frequency and percentages. Assessing the difference overtime in population-based and institutional-based c-sections was done through utilizing the binary variable of mode of delivery using the chi-square for trend and binary logistic regression after adjustment for all variables measured in the study. |  | |
| Statistical methods | 12 | (*a*) Describe all statistical methods, including those used to control for confounding  Frequencies and percentages of the measured variables were provided. Changes in proportion of institutional-based c-sections for each measured variable’s sub-category were tested by chi-square test for trend. Bivariate and multivariate logistic regression models were applied to assess the trend of c-sections, and the crude and adjusted associations of independent variables with the c-section delivery. |  | |
|  |  | (*b*) Describe any methods used to examine subgroups and interactions.  Not applicable |  | |
|  |  | (*c*) Explain how missing data were addressed.  Percentage of missing data for each category was reported in the tables. |  | |
|  |  | (*d*) If applicable, describe analytical methods taking account of sampling strategy  Sampling weights available in the EDHS databases was applied in all of performed statistical calculations. |  | |
|  |  | (*e*) Describe any sensitivity analyses  Not applicable |  | |
| Results | | |  | |
| Participants | 13* | **(a)** Report numbers of individuals at each stage of study—eg numbers potentially eligible, examined for eligibility, confirmed eligible, included in the study, completing follow-up, and analysed**.**  A total of 19,474, 16,527, and 21,762 women aged 15-49 years were randomly selected and surveyed in EDHS-2005, EDHS-2008, and EDHS-2014, respectively. The subjects of this study were restricted to mothers reported on the mode of birth delivery occurred five years prior to each survey round with a total weighted sample size of 29,107 from the three survey rounds. |  | |
|  |  | **(b)** Give reasons for non-participation at each stage.  Non-participation was mainly due to absence of household members at the survey time. |  | |
|  |  | **(c)** Consider use of a flow diagram  Flow diagram of selection of eligible study subjects was provided in figure 1. |  | |
| Descriptive data | 14* | **(a)** Give characteristics of study participants (eg demographic, clinical, and social) and information on exposures and potential confounders.  Tables 1 covered the characteristics of study participants |  | |
|  |  | **(b)** Indicate number of participants with missing data for each variable of interest.  Number of participants with missing data for each variable of interest was reported wherever its applicable in tables 1 and 2. |  | |
| Outcome data | 15* | Report numbers of outcome events or summary measures.  The only outcome of interest was c-section of birth delivery. Analysis revealed that the proportion of c-section deliveries rose substantially over the study period. |  | |
| Main results | 16 | (***a*)** Give unadjusted estimates and, if applicable, confounder-adjusted estimates and their precision (eg, 95% confidence interval). Make clear which confounders were adjusted for and why they were included.  Tables 3, 4, and 5 provide unadjusted and adjusted estimates for the variables under analysis with their 95% confidence intervals . |  | |
|  |  | (*b*) Report category boundaries when continuous variables were categorized.  Not applicable |  | |
|  |  | **(*c*)** If relevant, consider translating estimates of relative risk into absolute risk for a meaningful time period.  Not applicable |  | |
| Other analyses | 17 | Report other analyses done—eg analyses of subgroups and interactions, and sensitivity analyses  Not applicable |  | |
| Discussion | | |  | |
| Key results | 18 | Summarise key results with reference to study objectives  Analyses indicated a noticeable decline in home-based deliveries for a favor of institutional-based deliveries. There was a substantial rise in trend of institutional-based c-sections by more than three-folds, over the study period. This increase was primarily associated with significant increases among mothers of younger ages, mothers with low SES, primigravida or mothers with normal birth weight babies, and in the private sector. Although the private sector was the driver of the rising c-sections in Egypt, a substantial increase was also observed in use of this surgical procedure in public sector. The overtime discernible increase, by more than four-folds, in c-sections in the private sector was driven by substantial increases in c-sections among young, primigravida, or mothers reported normal birth weight babies. |  | |
| Limitations | 19 | Discuss limitations of the study, taking into account sources of potential bias or imprecision. Discuss both direction and magnitude of any potential bias.  The findings from the present study should be interpreted bearing the following limitations in mind. The cross-sectional design of the EDHS limits the causality pathway with regard to the drivers associated with increased c-sections. Given the nature of the household-based survey where medical records are usually unavailable, collected data did not include information about whether the c-sections were performed under medical indications such as fetal mal-presentation or based solely on maternal demand, except for the birth weight and birth multiplicity that served as the only obstetric indicators could potentially at medically necessary c-section. Despite of these limitations, the study provided evidence-based estimates on trend of c-section deliveries in Egypt and associated factors to fine-tune strategies necessary to halt the rising c-sections in Egypt. |  | |
| Interpretation | 20 | Give a cautious overall interpretation of results considering objectives, limitations, multiplicity of analyses, results from similar studies, and other relevant evidence.  The proportion of c-sections in Egypt has been increasing steadily in recent years and has reached an alarming level. The proportion of c-sections documented in the last EDHS conducted in 2014 quadrupled the maximum threshold recommended by the WHO. The increase in number of birth deliveries occurred in the private sector appears to be associated with a shift towards delivery in private facilities. This increase in the private sector, particularly among mothers who were potentially at low risk of c-sections requires an urgent need to adopt critical policies and strategies that able to halt the steady rise in c-sections in Egypt and improve reproductive health and mothers and babies health outcomes. In the meantime, an in-depth institutional-based study collecting data on the exact indications associated with c-sections in Egypt is also necessary. |  | |
| Generalizability | 21 | Discuss the generalizability (external validity) of the study results.  Findings assume that the observed significant rise in proportion of c-sections in Egypt is in line with global rise in c-sections that attributed to different drivers such as advancement of healthcare services, increasing access to healthcare services, and delayed maternal age. |  | |
| Other information | | |  | |
| Funding | 22 | Give the source of funding and the role of the funders for the present study and, if applicable, for the original study on which the present article is based  This work was supported by the Biostatistics, Epidemiology, and Biomathematics Research Core at the Weill Cornell Medicine‐Qatar. The statements made herein are solely the responsibility of the author. |  | |

*Give such information separately for cases and controls in case-control studies, and, if applicable, for exposed and unexposed groups in cohort and cross-sectional studies.

Separate versions of the checklist for cohort, case-control and cross-sectional studies are available on the STROBE website at www.strobe-statement.org
